# QuickPed: an online tool for drawing pedigrees and analysing relatedness

**DOI:** 10.1186/s12859-022-04759-y

**Published:** 2022-06-07

**Authors:** Magnus D. Vigeland

**Affiliations:** Department of Medical Genetics, Oslo University Hospital, Oslo, Norway

**Keywords:** Pedigree software, Pairwise relationships, Relatedness coefficients, Kinship, Inbreeding, Relatedness triangle

## Abstract

**Background:**

The ubiquity of pedigrees in many scientific areas calls for versatile and user-friendly software. Previously published online pedigree tools have limited support for complex pedigrees and do not provide analysis of relatedness between pedigree members.

**Results:**

We introduce QuickPed, a web application for interactive pedigree creation and analysis. It supports complex inbreeding and comes with a rich built-in library of common and interesting pedigrees. The program calculates all standard coefficients of relatedness, including inbreeding, kinship and identity coefficients, and offers specialised plots for visualising relatedness. It also implements a novel algorithm for describing pairwise relationships in words.

**Conclusion:**

QuickPed is a user-friendly pedigree tool aimed at researchers, case workers and teachers. It contains a number of features not found in other similar tools, and represents a significant addition to the body of pedigree software by making advanced relatedness analyses available for non-bioinformaticians.

**Supplementary Information:**

The online version contains supplementary material available at 10.1186/s12859-022-04759-y.

## Background

Drawing and analysing genealogical relationships are indispensable tasks in fields like medical genetics, forensic genetics, ecology and animal breeding, creating a demand for easily accessible software. Several free online tools for creating pedigrees are currently available, including *ped_draw* [1], *HaploForge* [2], *pedigreejs* [3], and *Progeny* [4]. However, these are geared towards clinical applications and have limited support for complex pedigrees commonly seen in areas like forensic genetics and animal breeding. For instance, all of the mentioned programs struggle with cross-generational mating (see Additional file 1: Fig. S1 for a simple example). Another limitation pertains to importing and exporting *ped files* describing pedigrees in text format. Such files are widely used to store pedigree data, both for purposes of reproducibility and for communication between software. Of the listed programs, *ped_draw* and *pedigreejs* import, but do not export, ped files. Conversely, *HaploForge* can save pedigrees as ped files after creation, but cannot import such files. The *Progeny* pedigree tool has no ped file support.

To the best of our knowledge, no online pedigree programs offer analysis of relatedness, like coefficients of kinship and gene identity. Such coefficients play an important role in many fields, as exemplified by recent studies in quantitative genetics [5], forensic genetics [6, 7] and ancient DNA [8]. Despite their widespread use, there is a serious lack of user-friendly software for computing relatedness coefficients, particularly for users without specialised bioinformatic skills.

X-chromosomal counterparts of the standard (autosomal) coefficients are easily defined and have a long history of applications, for instance in medical genetics [9] and forensic genetics [10]. However, it may be argued that the X-chromosomal coefficients remain considerably understudied, possibly due to the practical difficulties of computing them.

Here we introduce QuickPed, an interactive web tool for building and editing pedigrees, which also computes a wide variety of relatedness coefficients, both autosomal and X-chromosomal. In addition, QuickPed implements the *relatedness triangle* for visualising relatedness, and a novel algorithm producing verbal descriptions of pairwise relationships.

## Implementation

QuickPed is written in R using the *Shiny* package, and is powered by the *ped suite* packages for pedigree analysis in R [11]. In particular, the relatedness coefficients are computed with the *ribd* package [12], while the algorithm for describing relationships verbally descriptions, discussed in detail below, is implemented in *verbalisr*. Pedigrees are created with *pedtools* and plotted by importing *kinship2* [13], following standard pedigree nomenclature [14].

### Interactive pedigree creation

**Fig. 1:**
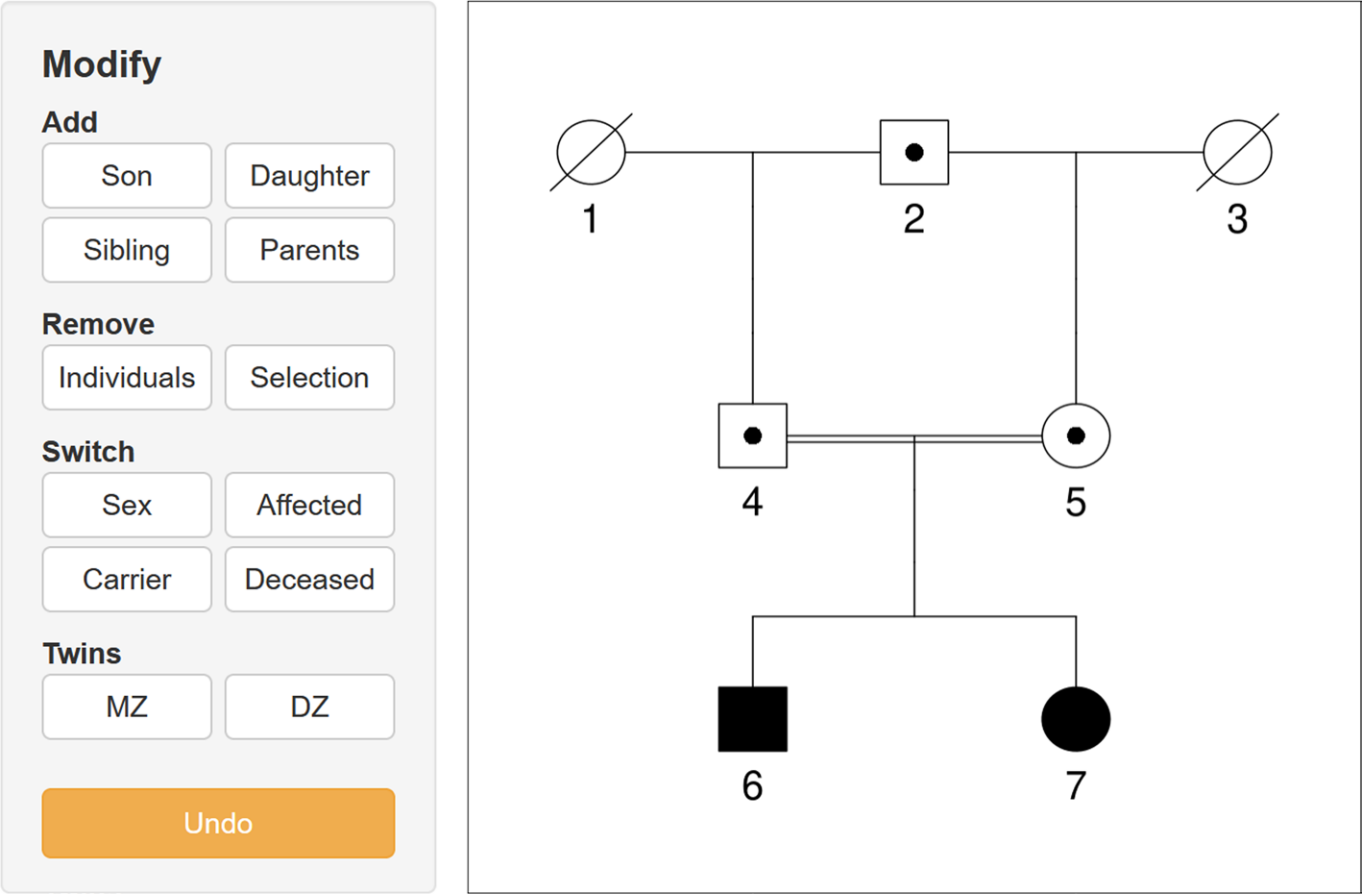
The pedigree editing frame of QuickPed, showing an example pedigree with various annotations. Individuals 6 and 7 are affected with some disease; 2, 4 and 5 are healthy carriers; 1 and 3 are deceased. The relatedness between 6 and 7 is analysed in the main text

To create a pedigree in QuickPed, the user can either choose one from the extensive built-in list or load an existing ped file. Malformed ped files are detected and generate informative error messages. Loaded pedigrees may be modified by selecting individuals and using appropriate buttons, as seen in Fig.1. The final result can be stored as an image (png or pdf) or as a ped file. Further instructions and information can be found at the QuickPed home page (see link below under Availability and requirements).

### Relatedness coefficients

Once a pedigree is created, a series of relatedness coefficients between its members can be computed. The following coefficients are supported, where *A* and *B* denote any members of the pedigree:

- The inbreeding coefficient $f_{\!A}$, defined as the kinship coefficient (see below) of the parents of *A*, or 0 if *A* is a founder [15].
- The kinship coefficient $\varphi _{\!AB}$, defined as the probability that a random allele from *A* and a random allele from *B* at the same autosomal locus, are *identical by descent* (IBD), i.e., that they have the same ancestral origin within the pedigree [15].
- The IBD coefficients $\kappa _{\!AB} = (\kappa _0, \kappa _1, \kappa _2)$, defined (for non-inbred individuals only) as the probability of sharing respectively 0, 1, or 2 alleles IBD at a random autosomal locus [16].
- The condensed identity coefficients $\Delta _{\!AB} = (\Delta _1, \dots , \Delta _9)$ of Jacquard [17].
- The detailed identity coefficients $\delta _{\!AB} = (\delta _1, \dots , \delta _{15})$ of Jacquard [17].
- X-chromosomal versions of all the above coefficients. Details about these can be found in the user manual.

For an introduction to these relatedness coefficients and their applications, see e.g., Thompson [18]. Lange [15] gives a more rigorous treatment with detailed algorithms, while Vigeland [11] focuses on calculations in R.

In addition to the standard coefficients described above, QuickPed also reports the relationship *degree*, as popularized by KING [19] and similar software for relatedness inference. In simple cases the degree equals the number of pedigree steps separating the individuals (e.g., 1 for parent-child and 2 for half siblings). More generally the degree is defined as a discretisation of the kinship coefficient $\varphi$, by rounding $\log _2(1/\varphi ) - 1$ to the nearest integer. This yields, for instance, degree 0 if $\varphi \in [\tfrac{1}{2}^{3/2}, 1] \approx [0.354, 1]$, degree 1 if $\varphi \in [\tfrac{1}{2}^{5/2}, \tfrac{1}{2}^{3/2}) \approx [0.177, 0.354)$, and degree 2 if $\varphi \in [\tfrac{1}{2}^{7/2}, \tfrac{1}{2}^{5/2}) \approx [0.088, 0.177)$.

For noninbred relationships, QuickPed implements a visualisation device known as the *relatedness triangle*, or *IBD triangle*. The IBD coefficients $(\kappa _0, \kappa _1, \kappa _2)$ of any such relationship can be viewed as a point $(\kappa _0, \kappa _2)$ in the plane triangle defined by $\kappa _0 \ge 0$, $\kappa _2 \ge 0$ and $\kappa _0 + \kappa _2 \le 1$ [11, 12]. The location of the most common relationships are indicated on the figure, as well as the inadmissible region established by Thompson [20], as a visual guide to the user.

### Relationship descriptions

QuickPed implements a novel algorithm for describing pairwise relationships, inspired by Wright’s path formula for the kinship coefficient [21]:

1 $$\begin{aligned} \varphi _{\!AB} = \sum _ {C, v_1, v_2} \frac{1}{2^{l_1 + l_2 + 1}}(1+f_C). \end{aligned}$$

The sum is over all common ancestors *C* of *A* and *B*, and all pairs $(v_1, v_2)$ of non-intersecting paths from *C* to *A* and *B*, respectively, with path lengths $l_1 = |v_1|$ and $l_2 = |v_2|$. Note that *C* may coincide with *A* or *B*, in which case the corresponding path has length 0.

To describe the relationship between *A* and *B*, the program first identifies all connecting paths, represented in the form $(C, v_1, v_2)$ as above, and classifies them as either *lineal* (if $l_1 = 0$ or $l_2 = 0$), *sibling* ($l_1 = l_2 = 1$), *avuncular* ($l_1 > l_2 = 1$ or *vice versa*) or *cousin* ($l_1, l_2 > 1$). Pairs of paths $(C, v_1, v_2)$, $(C', v_1, v_2)$ that are identical except that $C'$ is a spouse of *C*, are unified and tagged as *full*, while the remaining are *half*. The *path degree* is $l_1 + l_2 - \gamma$, where $\gamma$ is 1 if the path is full and 0 otherwise. For cousin paths we also define the *cousinship degree* as $\min (l_1, l_2) - 1$ and *removal* $|l_1 - l_2|$. Finally, the information about each path is translated to a human-readable statement in standardised format. Sets of paths with identical data $(l_1, l_2, \gamma )$ are reported together as *double* (or *triple*, etc.) relationships.

**Fig. 2:**
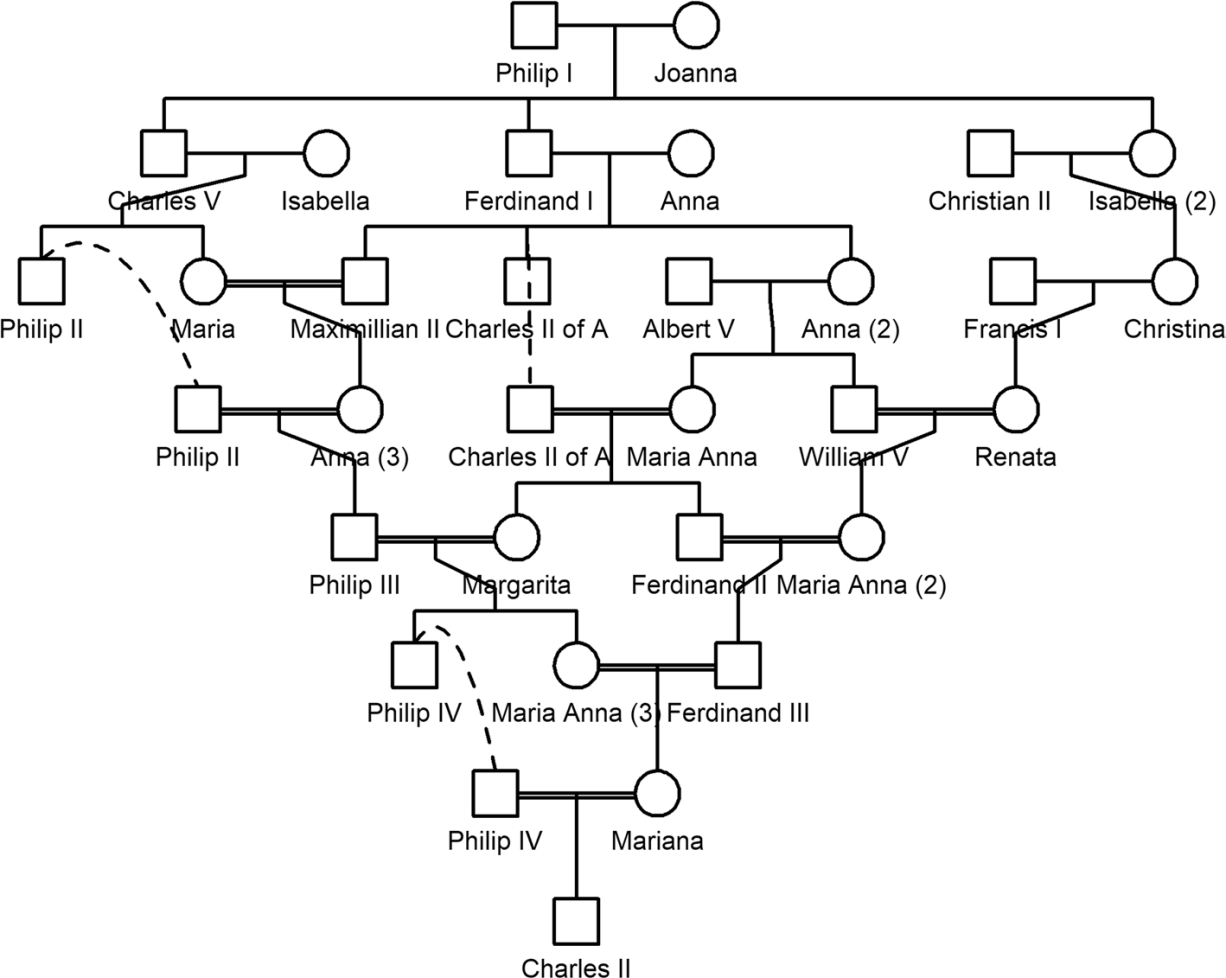
A branch of the Habsburg royal family, one of the historic pedigrees included in QuickPed

## Results

To illustrate the description algorithm, we consider the relationship between individuals 6 and 7 in Fig.1. They have four connecting paths, namely 6-[4]-7, 6-[5]-7, 6-4-[2]-5-7 and 6-5-[2]-4-7. In this notation, the ancestor *C* of each path is shown in brackets between $v_1$ and $v_2$. The first two paths merge into one full path, classified as *full siblings*. The two remaining paths both have $l_1 = l_2 = 2$, corresponding to half cousins of degree 1 with no removal. Being numerically equal they constitute a *double* relationship. The complete QuickPed output is as follows:

- Full siblings
  - 6-[4,5]-7
- Double half first cousins
  - 6-4-[2]-5-7
  - 6-5-[2]-4-7

For a more interesting demonstration, we applied the description feature to the famously complex pedigree of the Habsburg royalties. The inbreeding coefficient of King Charles II of Spain (1661–1700) has been estimated to approximately 0.25 [22], i.e., similar to that of a child produced by brother-sister incest. The ancestry of Charles II is included as one of the built-in pedigrees in QuickPed and reproduced in Fig.2. For Philip IV and Mariana (the parents of Charles II) the program reports that they are, simultaneously,

- Uncle-niece
- First cousins once removed
- Second cousins once removed
- Triple second cousins twice removed
- Triple third cousins
- Septuple third cousins once removed
- Sextuple third cousins twice removed
- Triple 4th cousins
- Septuple 4th cousins once removed

The complete pedigree paths are included in the output.

QuickPed offers a numerical summary of the selected relationship, by listing the standard relatedness coefficients. In the case of Philip IV and Mariana, we find:

- Inbreeding coefficients $f = 0.082$ and $f = 0.136$, respectively
- Kinship coefficient $$\varphi = 0.231$$
- Relationship degree $$= 1$$
- Identity coefficients $$\Delta = (0.011, 0.004, 0.044, 0.024, 0.077, 0.044, 0.071, 0.498, 0.228)$$

**Fig. 3:**
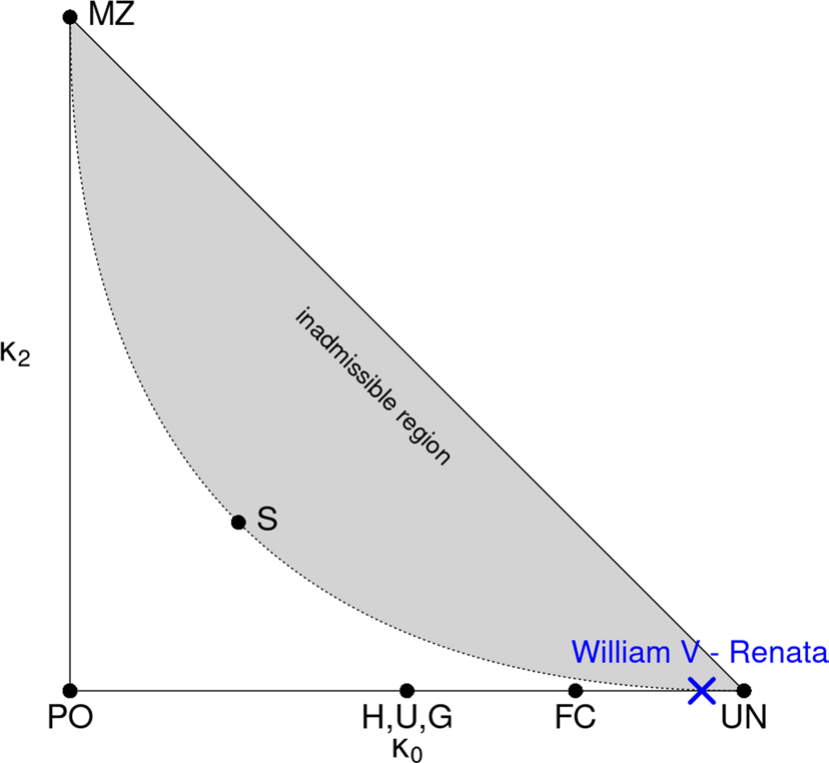
A relationship triangle showing the relationship between William V and Renata from Fig.2, in comparison with some common relationships. The triangle is drawn in the ($\kappa _0, \kappa _2)$-plane, each axis ranging from 0 to 1. Abbreviations: FC = first cousins; G = grandparent-grandchild; H = half siblings; MZ = monozygotic twins; PO = parent-offspring; S = siblings; U = uncle-nephew (and similar); UN = unrelated

Since Philip IV and Mariana are both inbred, their $\kappa$ coefficients are undefined. To exemplify the relatedness triangle, we therefore look at two other members of the Habsburg family, namely the second cousins William V and Renata (rightmost in the 4th generation). Fig.3 shows the point corresponding to their coefficients, $\kappa = (0.9375, 0.0625, 0)$, in comparison with other common relationships.

## Discussion

QuickPed aims to fill three gaps in the pedigree software literature. Firstly, it provides a quick, easy-to-use pedigree builder with robust support for import/export of ped files. Powered by the plotting abilities of *kinship2* [13], QuickPed supports many pedigrees which are poorly handled by comparable programs (Additional file 1: Fig. S1). Moreover, the interactive process is often accelerated by the many built-in templates, which includes both common pedigrees (e.g., aunt-nephew, first cousins), historic examples (e.g., Habsburg, Tutankhamun) and theoretically important relationships that are challenging to create from scratch (e.g., quadruple half first cousins). One limitation of QuickPed as a pedigree drawing program pertains to pedigree size. There is no hard-coded size limit, but in practice the plot window cannot comfortably display more than about 100 individuals. Another limitation is the set of annotation tools. For users requiring comprehensive clinical symbols we recommend *pedigreejs* [3] or *Progeny* [4].

Secondly, QuickPed is to our knowledge the first online calculator of pedigree coefficients. Particularly in the case of identity coefficients, existing programs like IdCoefs [23] demand nontrivial bioinformatic skills of the user, including a separate preparation of ped files. In QuickPed the entire process is interactive, making it more convenient for many users. Regarding X-chromosomal coefficients, we believe this to be an area of untapped potential, hindered by lack of software. It is our hope that QuickPed’s ability to calculate X-chromosomal versions of all available coefficients, including condensed and detailed identity coefficients, may stimulate some attention in this direction.

Finally, QuickPed introduces standardised descriptions of pairwise relationships. Although this feature was originally conceived for pedagogical purposes, we find that it has substantial practical merit. In the Habsburg family (Fig.2) it would be a daunting task to untangle the pedigree paths by hand. But also in much simpler cases, for example that in Fig.1, it is our experience that relationships are often specified imprecisely, even by specialists. As such, our algorithm provides a practical method to avoid misunderstanding and improve communication.

## Conclusion

QuickPed is a free, online pedigree tool primarily aimed at researchers, case workers and teachers. In addition to an intuitive pedigree builder, the program contains a variety of features for relatedness analysis, that are either novel or for the first time made accessible to non-bioinformaticians.

## Supplementary Information

**Additional file 1: Fig. S1** A pedigree with cross-generational mating, as displayed in various pedigree tools.** A** A ped file describing a pedigree with 5 individuals: Father (1), mother (2), daughter (3), son (4), and a child (5) resulting from father-daughter incest.** B** The pedigree as rendered by ped_draw [1], HaploForge [2], pedigreejs [3], and Progeny [4], respectively. For ped_draw and pedigreejs, the pedigree was loaded from the ped file, while HaploForge and Progeny required manual creation. In all cases, the result is inadequate.** C** The pedigree as shown in QuickPed.

## Data Availability

The Habsburg dataset analysed in this study is available as a built-in pedigree in QuickPed, and also in the source code repository: https://github.com/magnusdv/quickped/tree/master/data. Project name: QuickPed. Project home page (live app): https://magnusdv.shinyapps.io/quickped. Project information (user manual): https://magnusdv.github.io/pedsuite/articles/web_only/quickped.html. Project repository (source code): https://github.com/magnusdv/quickped. Operating system(s): Platform independent. Programming language: R. Other requirements: None. License: GPL3. Any restrictions to use by non-academics: Licence needed.

## Abbreviations

IBD: Identical by descent
