# Supplementary figures and images for: QuickPed: an online tool for drawing pedigrees and analysing relatedness
Source: BMC Bioinformatics. 2022 Jun 7;23:220. doi: 10.1186/s12859-022-04759-y (PMC9175388; doi:10.1186/s12859-022-04759-y)

A

| FAM | ID | FID | MID | SEX | AFF |
|-----|----|-----|-----|-----|-----|
| 1   | 1  | 0   | 0   | 1   | 1   |
| 1   | 2  | 0   | 0   | 2   | 1   |
| 1   | 3  | 1   | 2   | 2   | 1   |
| 1   | 4  | 1   | 2   | 1   | 1   |
| 1   | 5  | 1   | 3   | 1   | 1   |

B

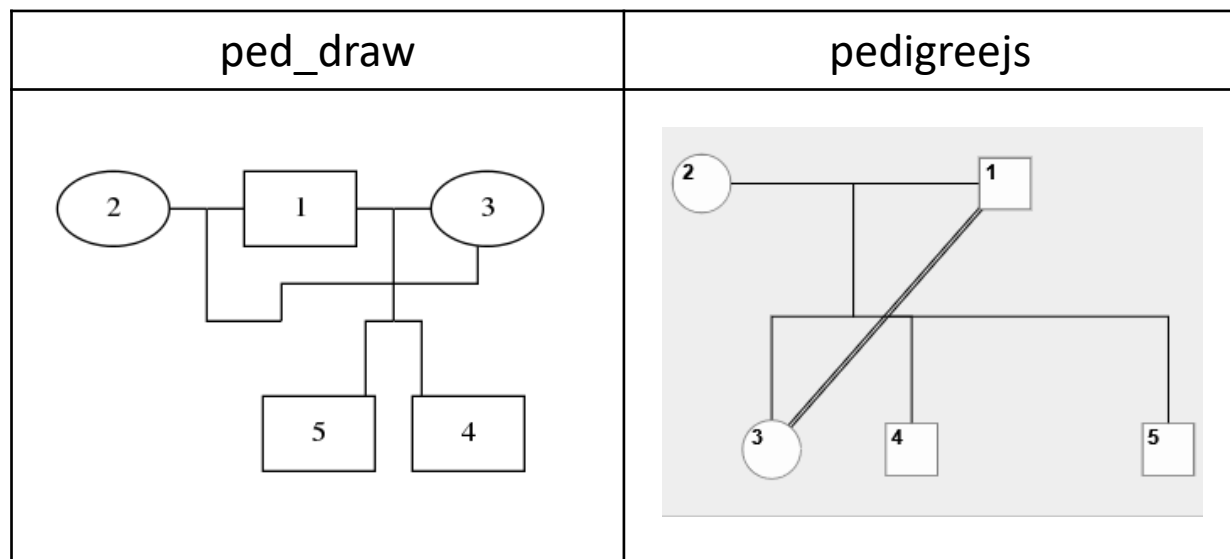

C

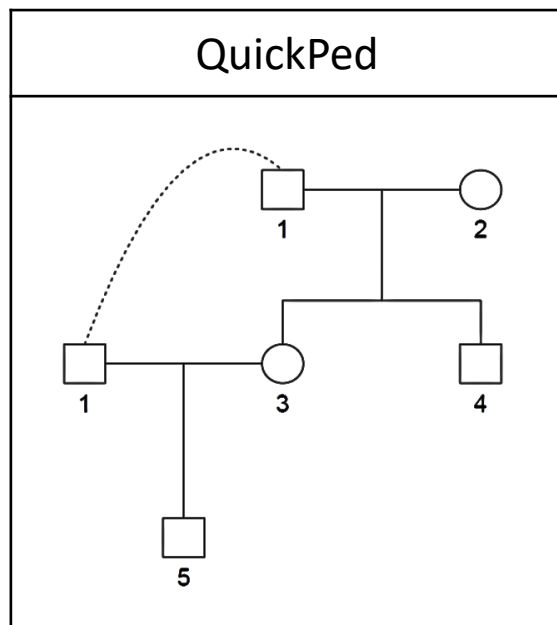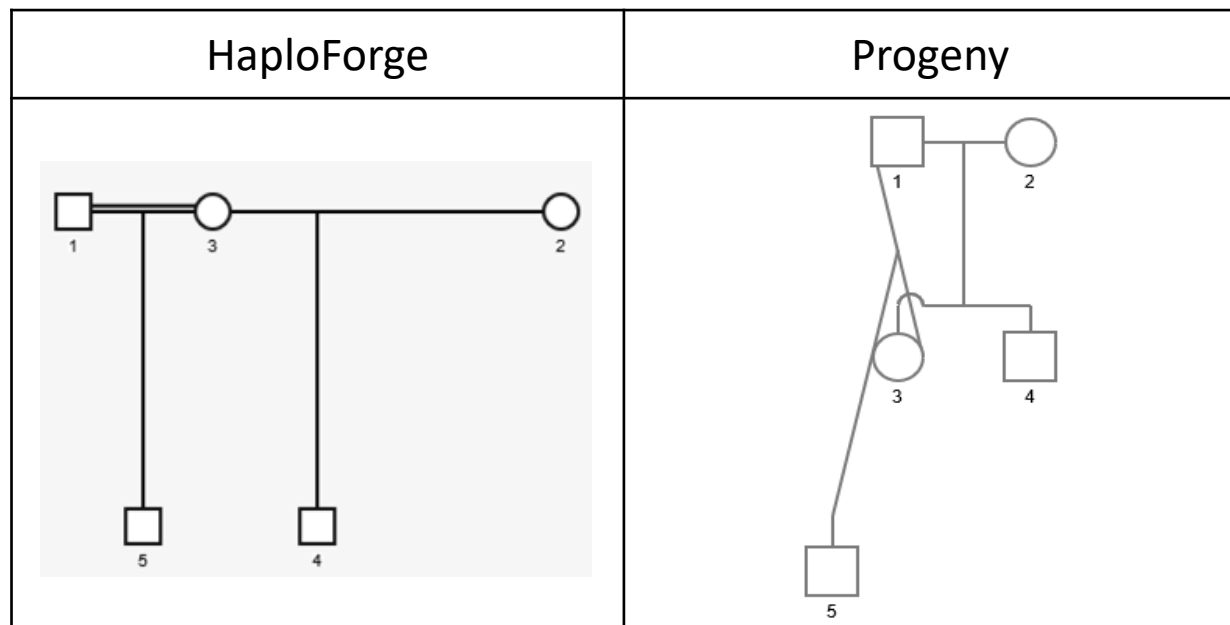

Supplement: Supplementary file 1 — Additional file 1: Fig. S1 A pedigree with cross-generational mating, as displayed in various pedigree tools. A A ped file describing a pedigree with 5 individuals: Father (1), mother (2), daughter (3), son (4), and a child (5) resulting from father-daughter incest. B The pedigree as rendered by ped_draw [1], HaploForge [2], pedigreejs [3], and Progeny [4], respectively. For ped_draw and pedigreejs, the pedigree was loaded from the ped file, while HaploForge and Progeny required manual creation. In all cases, the result is inadequate. C The pedigree as shown in QuickPed. [file 12859_2022_4759_MOESM1_ESM.pdf]
